# An Improved YOLOv5 Algorithm for Vulnerable Road User Detection

**DOI:** 10.3390/s23187761

**Published:** 2023-09-08

**Authors:** Wei Yang, Xiaolin Tang, Kongming Jiang, Yang Fu, Xinling Zhang

**Affiliations:** Mechanical and Vehicle Engineering, Chongqing University, Chongqing 400044, China; tangxiaolin6@126.com (X.T.); 20203201007@cqu.edu.cn (K.J.); 202132131099@cqu.edu.cn (X.Z.)

**Keywords:** improved YOLOv5 algorithm, overlapping targets, small targets, detection accuracy, AEBS-VRU system

## Abstract

The vulnerable road users (VRUs), being small and exhibiting random movements, increase the difficulty of object detection of the autonomous emergency braking system for vulnerable road users AEBS-VRUs, with their behaviors highly random. To overcome existing problems of AEBS-VRU object detection, an enhanced YOLOv5 algorithm is proposed. While the Complete Intersection over Union-Loss (CIoU-Loss) and Distance Intersection over Union-Non-Maximum Suppression (DIoU-NMS) are fused to improve the model’s convergent speed, the algorithm also incorporates a minor object detection layer to increase the performance of VRU detection. A dataset for complex AEBS-VRUS scenarios is established based on existing datasets such as Caltech, nuScenes, and Penn-Fudan, and the model is trained using migration learning based on the PyTorch framework. A number of comparative experiments using models such as YOLOv6, YOLOv7, YOLOv8 and YOLOx are carried out. The results of the comparative evaluation show that the proposed improved YOLO5 algorithm has the best overall performance in terms of efficiency, accuracy and timeliness of target detection.

## 1. Introduction

According to the most recent statistics provided by the Chinese National Bureau of Statistics, the number of road accidents has remained high over the past ten years. Each year, there are more fatalities involving vulnerable road users (VRUs), such as motorcyclists, bicyclists, and pedestrians. At the end of 2020, about 16,696 VRUs died from traffic accidents, accounting for 27.1% of the deaths that year. This number will show an upward trend with the continuous increase in the population and the complexity of traffic scenarios; therefore, the safety of VRUs has received an increasing amount of attention in recent years [1,2,3].

Faced with the special traffic conditions due to high traffic pressure and complex road target composition in China, the intelligent vehicle (IV) performance and requirements for AEB-VRUs are increasing. Since the VRUs’ behaviors are highly random, it is difficult to model their trajectories, and the system performance cannot be guaranteed in complex scenarios, such as “ghost probes” and intersections. A European prospect project study found that 16% of all vehicle accidents were caused by turning vehicles colliding with pedestrians at intersections [4]. Building an accurate VRU-aware algorithm that can effectively achieve collision avoidance in complex scenarios is one of the key technologies for automatic emergency braking systems (AEBSs), which has attracted extensive attention from researchers and recently turned into a hub for research.

With the development of artificial intelligence (AI) and computer vision (CV), deep-learning-based object detection is preferred. Currently, one-stage and two-stage detection algorithms make up the majority of deep-learning-based object detection techniques. The former creates a sparse collection of candidate boxes, classifies them, and then regresses them. The latter method directly pulls features from the source image to deduce the object’s location and label.

By employing the VGG-16 network and the Feature Pyramid Network (FPN) structure instead of AlexNet, Fast R-CNN [5] improves detection accuracy and speed. However, the operational redundancy arising from the selective search algorithm is still unavoidable, and because of that, the Fast R-CNN [6] algorithm is unable to satisfy the real-time demands of AEB-VRUs. Faster R-CNN [7] substitutes a Region Proposal Network (RPN) for a targeted search algorithm to lessen computational redundancy. Its mean average precision (mAP) can reach 73.2% on the Pattern Analysis, Statical Modeling and Computational Learning Visual Object Classes (PASCAL VOC) 2007 dataset, but its detection speed is only five frames per second (FPS), which is still insufficient to match its real-time requirements. He et al. [7] presented a mask Region-based K Nearest Neighbors (R-KNN) algorithm that improved Faster R-CNN using instance segmentation, but the detection accuracy and speed did not improve significantly.

The single-stage detection algorithms omit the candidate region generation stage, extract features directly from the image based on global information, and after that, determine the object’s categorization and location. The Single Shot MultiBox Detector (SSD) algorithm and the You Look Only Once (YOLO) algorithm are representative of the single-stage algorithms and have been widely applied in object detection. Berg et al. [8] designed the SSD algorithm taking advantage of the YOLO algorithm and Faster R-CNN algorithm, which obtained six different semantic levels of feature maps for classification and regression with down-sampling. The SSD algorithm can achieve a multi-scale fusion effect, and its detection accuracy and speed are relatively balanced. Nevertheless, it is ineffective for classifying small objects and lacks deep semantic information. To overcome its weaknesses, algorithms like Deconvolutional SSD (DSSD) [9], Rainbow SSD (RSSD) [10], and Feature Fusion SSD (FSSD) [11] were suggested to enhance the robustness of detection of small objects.

In series of YOLO algorithms, each image is split into S × S grid cells by the YOLO algorithm [12], with each grid only accountable for predicting the items whose center points fall within it. Consequently, the YOLO algorithm can infer the specific location and label information of all objects only once to realize end-to-end training and forecasting, and the detection speed can reach 45 FPS. Redmon et al. [13] proposed the YOLOv2 algorithm with the two-stage detection as a reference framework in order to reduce the YOLO algorithm’s lack of precision for small-scale object localization. This algorithm introduced the Anchor Box mechanism and employed DarkNet-19 [14] as the backbone network, and calculated the anchor position through cluster analysis to significantly improve the precision of the detection and recall rates. However, the detection precision of YOLOv2 on small and overlapping objects was still low owing to the poor positioning accuracy of the bounding box. Redmon et al. [15,16] presented the YOLOv3 algorithm to perform bounding box prediction by adding multi-scale prediction capabilities and using deeper DarkNet-53 and pyramid networks. Compared to YOLOv2, YOLOv3 had greater accuracy, but its detection speed did not improve significantly due to the deeper network structure.

By including CutMix and Mosaic data improvement in the input port and replacing DarkNet-53 with the Cross Stage Partial (CSP)-DarkNet53 as the backbone, Bochkovskiy et al. [17] developed the YOLOv4 algorithm [18]. To increase the model’s perceptual field, the Spatial Pyramid Pooling (SPP) module was introduced to the neck network, and multi-channel feature fusion was accomplished using the path aggregation network (PAN). By including new features like adaptive anchor picture calculation and adaptive picture resizing in the input, Jocher et al. [19] introduced the YOLOv5 algorithm, with a backbone network that merged the Focus structure and CSP structure to achieve the slicing operation. Thus, the network feature fusion capability and the detection performance were further enhanced in the YOLOv5 algorithm.

The two-stage algorithm based on candidate region extraction has high detection accuracy for targets, and it can achieve accurate detection for targets with high overlap density and small targets. Nevertheless, its biggest drawback is low detection efficiency. Selective search algorithms result in slow network feature extraction, long training and detection processes, and cannot meet the real-time requirements of intelligent vehicle environment perception. The model structure of the single-stage algorithm is agile, with high real-time detection efficiency, and its detection rate and accuracy are balanced. The existing YOlO5 original model has deep feature maps and is not easily able to learn the feature information of small target samples such as pedestrians. As such, there is a requirement to improve the YOlO5 algorithm. In the input layer, a four-time sampling process is applied to the input image in the YOLOv5 input end. This results in new-sized feature maps in the feature fusion network, which have smaller receptive fields and richer positional information. This effectively improves the detection performance for small target groups such as VRUs.

- In the backbone layer, before the image enters the feature extraction network, a process is applied to obtain four sets of images with similar morphologies and no information loss by sampling every other pixel in each image. These images are then fed into the input channels, expanding the width and height information of the images by four times. This increases the receptive field of the model during convolutional operations, resulting in more comprehensive feature extraction and obtaining down-sampled feature maps that are twice as large.
- In the neck layer, a solution method is introduced to address the issue of only transmitting semantic information without conveying localization information in the Feature Pyramid Network (FPN). The Perceptual Adversarial Network (PAN) is incorporated, forming the “FPN + PAN” structure, which enables the transmission of strong localization features from shallow layers to deeper layers. Additionally, the neck layer also incorporates the CSP2 structure, inspired by the design of CSPNet, to further enhance the feature fusion capability of the network model.
- In the prediction layer, we propose a modified calculation method for the GIoU-Loss function, which take into account the shape of the rectangles. Additionally, in the post-processing stage of the model, the DIoU-NMS method is introduced to improve the object box filtering mechanism, making it easier to detect small and overlapping objects. This addresses the issue in the original YOLOv5, where the Weighted Non- Maximum Suppression (WNMS) algorithm used to remove redundant predicted boxes had poor suppression effects in the presence of occluded or overlapping objects.

The following is the structure of this paper. First, the network structures of various YOLOv5 versions are introduced, and the foundational model for target detection is selected as the YOLOv5s model. The YOLOv5s network architecture is improved from four aspects: input port, backbone network, neck network, and prediction port. Second, vulnerable road groups such as pedestrians, cyclists, and motorcyclists, as well as major groups that pose a safety threat to VRUs, such as passenger cars, trucks, and buses, are used as key detection targets. Suitable images are selected and labeled in the nuScenes, Caltech, and Penn-Fudan datasets as training and test sets for the improved YOLOv5 algorithm. The above datasets are used to create the training and test sets. Third, the improved YOLOv5s algorithm is trained using transfer learning, and the detection precision and speed are compared using Faster R-CNN, YOLOv3, YOLOv6, SSD, and YOLOv5 algorithms on the same dataset. Last, the detected object results are discussed and conclusions are presented.

## 2. YOLOv5 Algorithm

**Figure 1:**
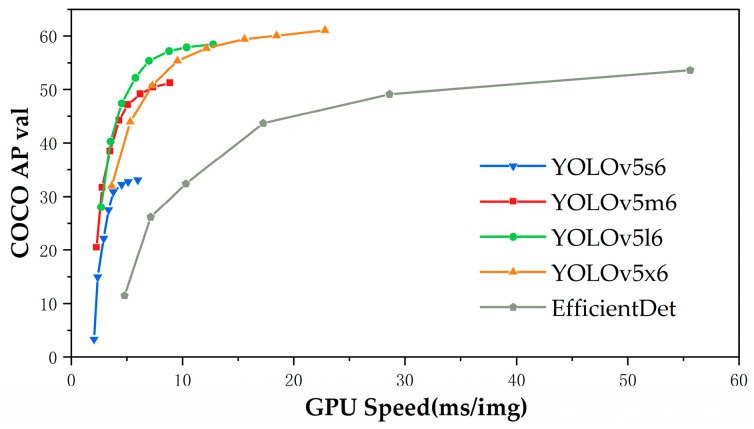
Comparison of various YOLOv5 models [20].

Figure 1 shows the four variations of the YOLOv5 algorithm: YOLOv5s, YOLOv5m, YOLOv5l, and YOLOv5x. The smallest and fastest network model, shallowest depth, and shortest feature map width among these algorithms are all found in YOLOv5s. Thus, considering the detection accuracy and mobile online requirements, the YOLOv5s algorithm is chosen as the basic framework for object detection.

The YOLOv5 algorithm firstly preprocesses the input images at the input layer, including the automatic calculation of the optimal anchor size based on the iterative K-means clustering of the genetic algorithm, as well as the enhancement of the Mosaic data by four-in-one adaptive stitching and scaling of the four random images in the training set. In the detection process, the algorithm divides the input tensor into S × S grids. If the center point of the target located in a grid, the grid is responsible for the detection of the target. For each grid, predict B anchors on it. Specifically, for each anchor box, predict (5 + C) values. The first five values are used to regress the central point position of the anchor frame and the size of the frame, and to determine whether there is a target in the frame; they are: the distances *t*_x_ and *t*_y_ between the center coordinates of the prediction frame and the coordinates of the upper left point of the grid, the scaling coefficients *t_w_* and *t_h_* of the width and height of the prediction frame and the anchor frame, and the confidence degree Conf to judge whether there is a target. *C* is the total number of target categories. If the box contains targets, the extracted targets are classified. The conversion formula between the predicted value and the output target position is as follows [21]:

(1) $$x=\sigma (t_{x})+c_{x}$$

(2) $$y=\sigma (t_{y})+c_{y}$$

(3) $$w=a^{w}e^{t_{w}}$$

(4) $$h=a^{h}e^{t_{h}}$$

where *a^w^* and *a^h^* are the width and height of the anchor frame, and *c_x_* and *c_y_* are the coordinates of the upper left point of the grid where the anchor frame is located.

For the regression of the target center position, the Sigmoid (*σ*) function is used to map the offset between the center coordinates predicted by the network and the coordinates of the upper left point of the grid where the center point is located to [0,1]. By virtue of the processing of the Sigmoid function, the center position of the prediction box will be constrained inside the current grid, limiting the amplitude of the deflection. Add the mapped offset to the top left coordinates *c_x_* and *c_y_*, and finally obtain the center coordinates (*x*, *y*) predicted by the network.

The total loss is:

(5) $$l_{box}=\lambda_{coord}\sum_{i=0}^{S^{2}}\sum_{j=0}^{B}I_{i,j}^{obj}\left(2-w_{i}\times h_{i}\right)[{\left(x_{i}-{\overset{\wedge }{x}}_{i}^{j}\right)}^{2}+{\left(y_{i}-{\overset{\wedge }{y}}_{i}^{j}\right)}^{2}+{\left(w_{i}-{\overset{\wedge }{w}}_{i}^{j}\right)}^{2}+{\left(h_{i}-{\overset{\wedge }{h}}_{i}^{j}\right)}^{2}]$$

(6) $$l_{cls}=\lambda_{class}\sum_{i=0}^{S^{2}}\sum_{j=0}^{B}I_{i,j}^{obj}{\sum_{c\in classes}P}_{i}\left(c\right)\log(\overset{\wedge }{P_{i}}(c))$$

(7) $$l_{obj}=\lambda_{noobj}\sum_{i=0}^{S^{2}}\sum_{j=0}^{B}I_{i,j}^{noobj}{\left(c_{i}-\overset{\wedge }{c_{i}}\right)}^{2}+\lambda_{obj}\sum_{i=0}^{S^{2}}\sum_{j=0}^{B}I_{i,j}^{obj}{\left(c_{i}-\overset{\wedge }{c_{i}}\right)}^{2}$$

(8) $$Loss=l_{box}+l_{cls}+l_{obj}$$

where *a^w^* and *a^h^* are the width and height of the anchor frame, *c_x_* and *c_y_* are the coordinates of the upper left point of the grid where the anchor frame is located, *l_box_* is the position regression loss and *λ_coord_* is the position loss coefficient, *l_cls_* is the class loss and *λ_cls_* is the class loss coefficient, $\overset{\wedge }{x}$ and $\overset{\wedge }{y}$ are the true center coordinates of the target, and  $\overset{\wedge }{w}\;\mathrm{and}\;\overset{\wedge }{h}$ are the width and height of the target. If the anchor box at (*i*, *j*) contains the target, the value of $I_{i,j}^{obj}$ is 1; otherwise, the value is 0. $p_{i}(c)$ represents the class probability of the target, $\overset{\wedge }{p_{i}}(c)$ is the true value of the class, and the length of both is equal to the total number of classes *C*.

## 3. Improved YOLOV5 Algorithm

**Figure 2:**
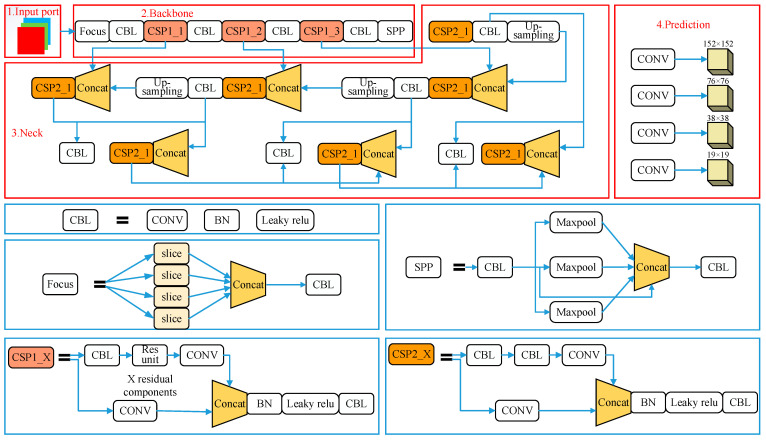
Architecture of the improved YOLOv5s model.

The improved YOLOV5 algorithm is based on the YOLOv5s algorithm, which mainly focuses on the network structure and loss function. Figure 2 depicts the structure of the developed YOLOv5s network in detail, involving the input port, backbone layer, neck layer, and prediction layer, which make up the YOLOv5s model.

### 3.1. Input Port

The input port of YOLOv5 includes Mosaic data enhancement, adaptive anchor frame calculation, and adaptive picture scaling. Referring to the CutMix method [22], the Mosaic data enhancement considerably improves the algorithm’s recognition of small target groups, such as those in VRUS, by stitching four photos in random scaling, random cropping, and random configurations. Furthermore, the robustness of the model is improved, while the memory requirement is decreased.

The input image’s dimensions are set to 608 × 608. The size scaling and the black edge filling methods are employed to standardize the image size during the training process, and the adaptive black edge filling algorithm is used during the testing process. After calculating the scaling ratio and the scaling factor, images with different sizes are filled to the standard sizes to decrease the time spent on testing the model. The model generates the prediction box according to the initialized anchor box during the model training process. The best anchor box in the training set can be determined adaptively at the same time as the size of the anchor is updated based on the loss after comparing it with the ground truth box. Hence, the initial placement of the anchor box is a crucial aspect of the model training. The large-scale feature map should be regressed with a small-sized anchor box for the prediction box since the smaller the perceptual field, the larger the feature map.

### 3.2. Backbone Layer

**Figure 3:**
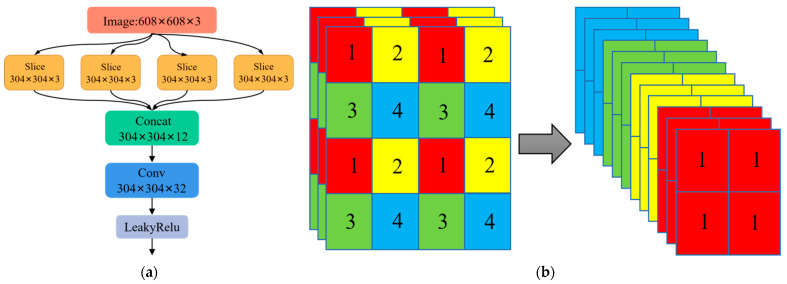
Architecture of the “Focus” network and the principle of the slice operation. (**a**) Architecture of the Focus network structure; (**b**) schematic diagram of the slice operation process.

The most of the backbone layer is made up of the Focus structure and CSP-Darknet53. Figure 3a shows the Focus network structure. The images are sliced prior to feature extraction according to the slice operation process shown in Figure 3b. In each image, we consider the value of one pixel at each interval to obtain four sets of images with similar morphologies and no loss of information. Subsequently, the four sets of images are fed into the input channels. Thus, the image’s width and height are increased by 4, and the quantity of channels is expanded to 12. Finally, the convolution operation is carried out to broaden the model’s sensory range of view, and a two-fold down-sampling feature map with fuller feature extraction is obtained.

### 3.3. Neck Layer

**Figure 4:**
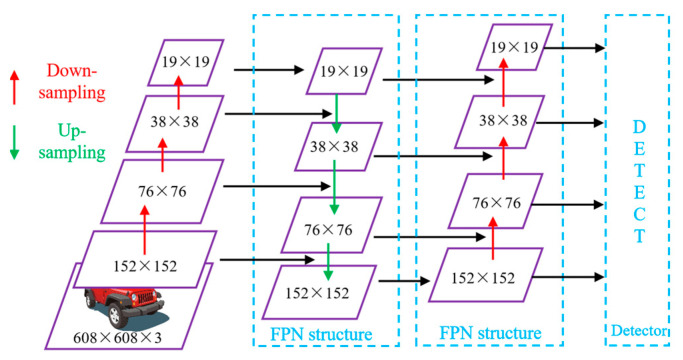
Architecture of the improved “FPN + PAN”.

The neck layer is employed to generate an FPN that enhances the inference of the model for objects at different scaling factors. The shallow Feature Map is the inverse of the deep Feature Map in that it has lower localization information and richer semantic features. The FPN enhances the semantic representation of the entire pyramid network at all sizes by switching the strong semantic properties from the deep layer to the shallow layer. It does not carry localization information; only semantic information. In order to move the strong localization features from the shallow layer to the deep layer, the PAN [23] adds a bottom-up inverted pyramid structure behind the FPN, creating the “FPN + PAN” twin-tower construction shown in Figure 4. In addition, the CSP2 structure, which is based on the CSPNet design, is added to the neck layer to increase the capacity of the network model for feature fusion.

### 3.4. Prediction Layer

The target detection algorithm frequently uses intersection over union (IoU) as a metric to assess the Bounding Box regression loss. The range of IoU is [0,1], it has scale invariance, and it can measure the matching degree of various shapes. Suppose *B* is the prediction Bounding Box, $B^{gt}$ is the true box Ground Truth, and $B\cup B^{gt}$ denotes the concatenation of two boxes. The expression of IoU is

(9) $$IoU=\frac{\left|B\cap B^{gt}\right|}{\left|B\cup B^{gt}\right|}$$

**Figure 5:**
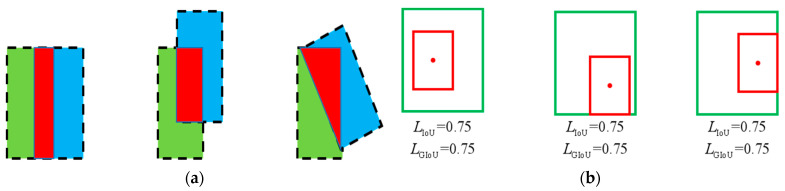
Failure scenarios of IoU and GIoU loss. (**a**) IoU failure; (**b**) GIoU failure.

When the loss function is used to define IoU, the calculation process is simple and the training and inference are fast; however, it does not fully reflect the overlapping shape between the predicted and actual boxes. For example, the IoU values are the same in the three cases shown in Figure 5a, but the specific overlap is different. In addition, when there is no intersection between the two frames, IoU=0 results in gradient disappearance, which makes it impossible to backpropagate and train the deep learning network. These drawbacks can be avoided using the generalized intersection ratio loss function Generalized IoU (GIoU)-Loss. The following is how Generalized IoU (GIoU)-Loss is expressed [24]:

(10) $$L_{GIoU}=1-IoU+\frac{\left|C-B\cap B^{gt}\right|}{\left|C\right|}$$

where *C* stands for the detection frame’s and the real frame’s minimum enclosing rectangle.

The advantage of GIoU is that it uses the minimal closed form to ensure that the training gradient remains visible even in the absence of a detection frame–real frame intersection. Nevertheless, in cases such as the inclusion shown in Figure 5b, the training can continue, and the prediction frame can be approximated as close as possible to the real frame without overlapping.

The distance cross-merge ratio loss function DIoU-Loss and the complete cross-merge ratio loss function CIoU-Loss are combined [25] to solve the issue of IoU and GIoU. The DIoU-Loss is calculated as

(11) $$L_{DIoU}=1-IoU+\frac{\rho^{2}\left(B,B^{gt}\right)}{c^{2}}$$

**Figure 6:**
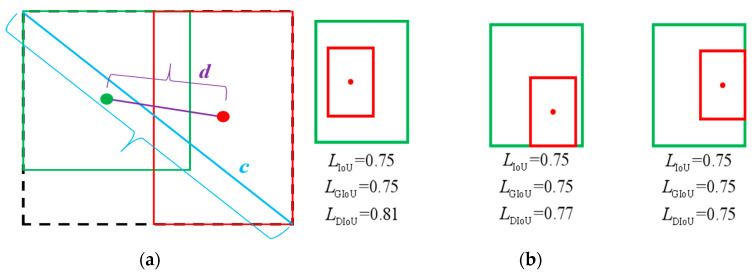
Illustration of IoU, GIou and DIoU. (**a**) Calculation diagram of DIoU; (**b**) loss comparison among IoU, GIoU, and DIoU.

As Figure 6a shows, the DIoU-Loss introduces the Euclidean distance d between the center point of the predicted and real frames, and the diagonal length *c* of the smallest external rectangle between the predicted and real frames. It achieves a faster training speed and solves the problem of GIoU-Loss by optimizing the distance between the two frames. Figure 6b shows the comparison of the three IoU loss calculations, and it can be observed that the DIoU-Loss better represents the true overlapping shape of the detected frame and the real frame.

However, the above three IoU-Loss calculation methods do not consider the specific shape of the rectangular frame. Three geometrical elements are examined by the CIoU-Loss: the overlap region between the detection and real frames, the separation from the center point, and the aspect ratio. This can render the prediction frame more reliable in the course of the regression process and give a higher convergence accuracy. Accordingly, it is suggested that the GIoU-Loss function be replaced with the CIoU in the YOLOv5 model. As formula (12) shows, the CIoU-Loss is calculated by adding the influence factor to the DIoU-Loss, where αv denotes the weight parameter, v stands for the difference between the width-to-height ratio of the prediction frame and the real frame, and w and h are the width and height of the detection frame, respectively. When there is no difference between the prediction frame and the real frame, v=0.

(12) $$L_{CIoU}=1-IoU+\frac{\rho^{2}\left(B,B^{gt}\right)}{c^{2}}+\alpha v,\;\alpha =\frac{v}{1-IoU+v},\;v=\frac{4}{\pi^{2}}{\left(\arctan\frac{w^{gt}}{h^{gt}}-\arctan\frac{w}{h}\right)}^{2}$$

## 4. Deep Learning Target Detection Datasets

The AEB-VRU system focuses on vulnerable road groups like pedestrians, bicyclists, and motorcyclists, whereas passenger cars, trucks, and buses are the main groups that pose safety threats to the VRUs. Given the characteristics of massive car ownership in China and complex road traffic flow, suitable images from the public datasets listed in Table 1 were selected and labeled as the training and test sets for the improved YOLOv5 algorithm, respectively.

The most common image annotation formats are Extensible Markup Language (XML) in PASCAL VOC and txt in the YOLO series algorithm. The ground truth information was generated in the XML format using Labeling software, and the program to convert the XML format to text format was written in Python. The normalized target frame width *w*, normalized target frame height *h*, normalized center point *x*, normalized center point *y*, and the category of the annotation content were all contained in the text.

**Figure 7:**
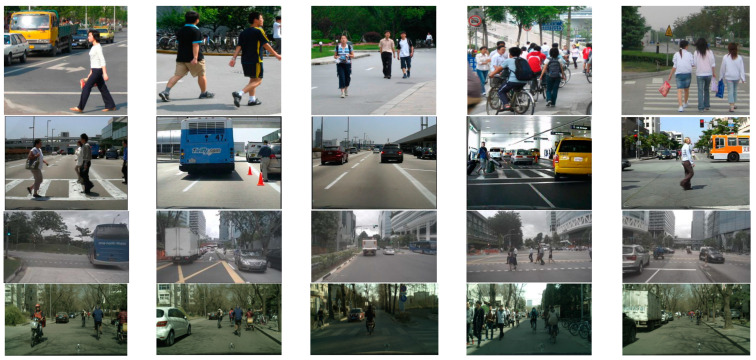
Some of the VRU dataset images.

After image screening and processing data, about 5000 images were selected as the VRU dataset for the improved YOLOv5 algorithm, some of which are shown in Figure 7. The code to convert the format from XML to text and automatically divide the set used for training from the dataset was written in Python. The ratio of the training to test set was 8:2.

## 5. Training Methods and Evaluation Indicators

**Figure 8:**
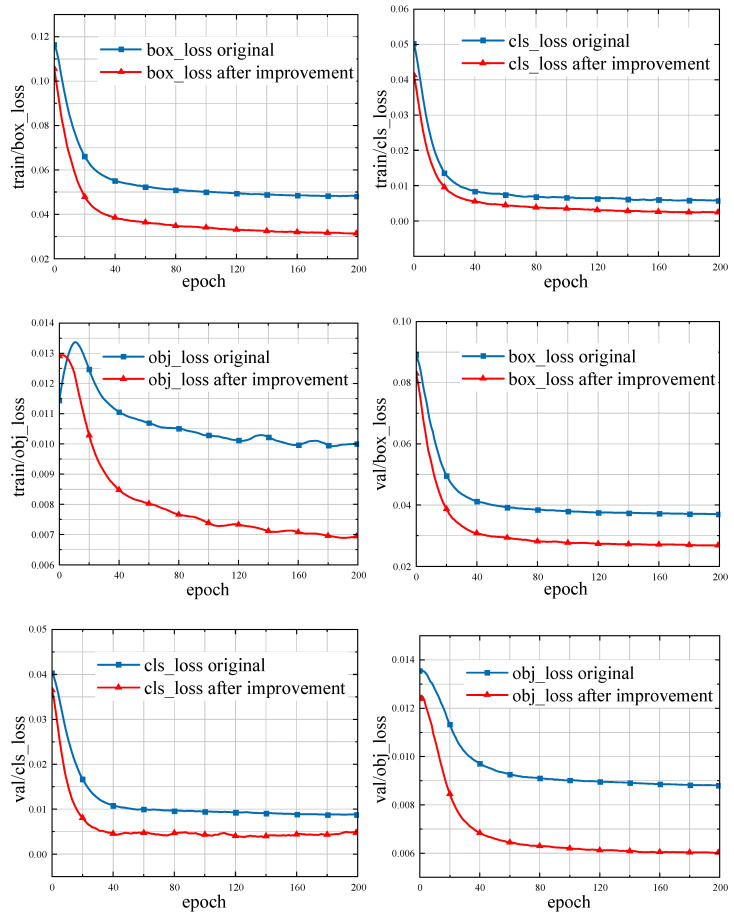
Loss curve of the training process.

The dataset used in this research has some similarities with the COCO dataset, which was used to train the original YOLOv5 algorithm [15]. Therefore, the improved YOLOv5s algorithm was trained using transfer learning to increase the training effectiveness and hasten the convergence of the YOLOv5 model. Table 2 shows the allocation of computational resources during training. It took 10.5 h for the model training to be completed. The change in training loss in Figure 8 (smoothed data) shows that the adoption of migration learning training can accelerate model convergence. Furthermore, the regression loss (box_loss), confidence loss (cls_loss), and category loss (obj_loss) of the improved algorithm are further reduced in the training and validation sets.

Precision (P), Recall (R), Average Precision (AP), and mAP are the primary measures used to assess the effectiveness of the target detection algorithms across all categories. They are calculated as shown in (13)–(16), where TP means True Positive, FP means False Positive, and FN means False Negative.

(13) $$P=\frac{TP}{TP+FP}$$

(14) $$R=\frac{TP}{TP+FN}$$

(15) $$AP=\int_{0}^{1}P(R)dR$$

(16) $$mAP=\frac{1}{classes}\sum_{i=1}^{classes}AP_{i}$$

**Figure 9:**
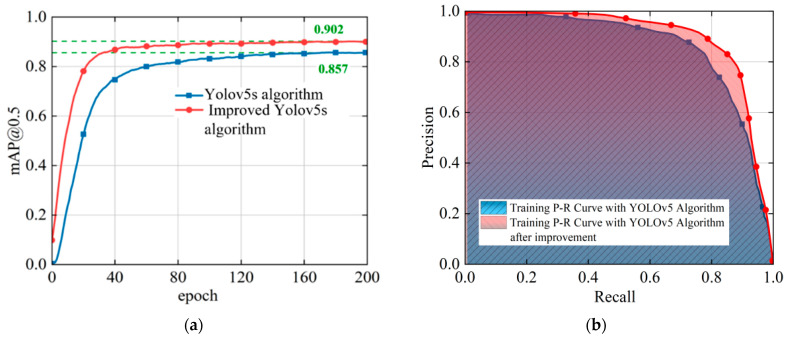
Comparison between the original YOLOv5 algorithm and the improved YOLOv5 algorithm. (**a**) P-R curve comparison; (**b**) mAP curve comparison.

The P-R curves and mAP curves of the two models are plotted individually to more clearly reflect how the improved YOLOv5 algorithm performs better than the original YOLOv5 algorithm, in Figure 9. The P-R curve of the improved YOLOv5 algorithm shown in Figure 9a fully envelopes the P-R curve of the original YOLOv5 algorithm, which indicates that the improved algorithm further balances the precision and recall rates, and the performance is significantly improved. Figure 9b depicts that the average precision of all class labels mAP@0.5 is 90.2%, increasing 4.5% compared to 85.7%, which was obtained using the original algorithm, and enhancing the detection performance for small-scale objects such as overlapping pedestrians and cyclists.

**Figure 10:**
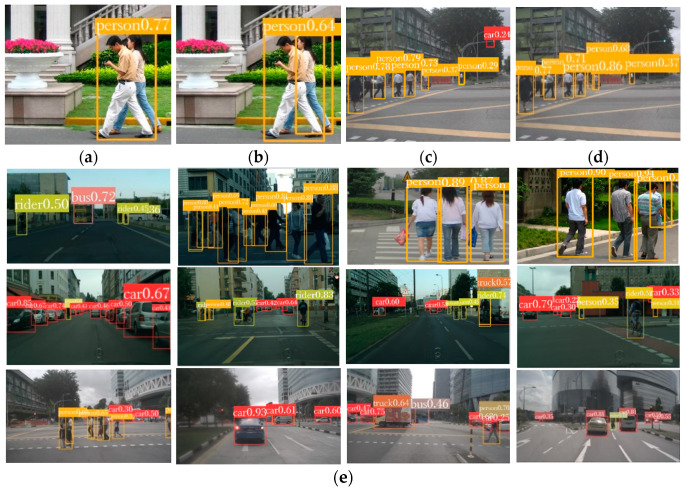
Partial detection exhibition of the improved YOLOv5 algorithm. (**a**) Detection performance of original YOLOv5 algorithm; (**b**) improved detection performance; (**c**) detection performance before improvement; (**d**) improved detection performance; (**e**) detection performance for other parts of the scene.

Some detection performance results of the improved YOLOv5 algorithm are shown in Figure 10. Different objects are effectively detected in complex traffic scenarios composed of pedestrians, vehicles, cyclists, etc. This implies that our work has greatly improved the performance of detection for small objects. There are few cases of skipped or incorrect detection, showing that the algorithm is appropriate for AEB-VRUs.

The algorithm also performs well in special scenes such as vehicle occlusion and pedestrian overlap, and it achieves a high detection confidence level, which demonstrates the robustness and generalization ability of the improved YOLOv5 algorithm. More importantly, the YOLOv5 algorithm has a very high detection speed.

## 6. Improved Algorithm Results and Discussion

The original algorithm obtained three different feature maps of sizes 76 × 76, 38 × 38 and 19 × 19 using down-sampling, that is, 8, 16, and 32 times, respectively. In the input port of the improved YOLO5, a four-time down-sampling process is used in the network entering the feature fusion network to obtain an updated size map feature of size 152 × 152. The accuracy of detection for small items like VRUs can be significantly improved by the feature map’s reduced receptive field and better position information. The more types of feature maps there are, the more grids and anchor boxes the image is divided into, making it easier to detect smaller or overlapping targets. This improves detection ability while slightly reducing inference speed.

In backbone layer of the structure of the proposed algorithm, the CSPDarkNet53, which is the core of the algorithm, used to extract target features, is divided into two structures: the CSP1 structure for the backbone network and the CSP2 structure for the neck network. The base layer’s feature mapping is split into two sections by the CSPNet before being combined using a cross-stage hierarchy. Based on this, the duplication of network optimization gradient information in other convolutional neural networks integrates gradient changes into the feature map, which improves the capacity of convolutional neural networks for learning. The CSPNet can ensure a lightweight structure without any accuracy loss, and it can also reduce computational complexity.

Replacing the GIoU-Loss function in the YOLOv5 model with the CIoU-Loss function with a better theoretical effect in the BOX section of the calculation loss function improves the model’s detection performance and robustness. With the GIoU-Loss in the original YOlO5, when the target box completely contains prediction boxes of the same size, the function value remains unchanged no matter how the position of the prediction box changes. Aiming at overcoming this drawback, considering the relative position, center point distance, and aspect ratio of the two boxes, an influence factor has been used in the CIoU-Loss function calculation formula, which can more accurately reflect the loss situation during model training.

In addition, the Weighted Non-Maximum Suppression (WNMS) algorithm does not perform well for occlusions and overlapping objects during the removal of redundant prediction frames in the post-detection processing of the original YOLOv5 algorithm. The objects of the AEB-VRU system are mainly pedestrians, cyclists, and other VRUs, which are prone to occlusion. Consequently, the proposed algorithm can significantly improve the object frame screening mechanism and easily detect small and overlapping objects.

**Figure 11:**
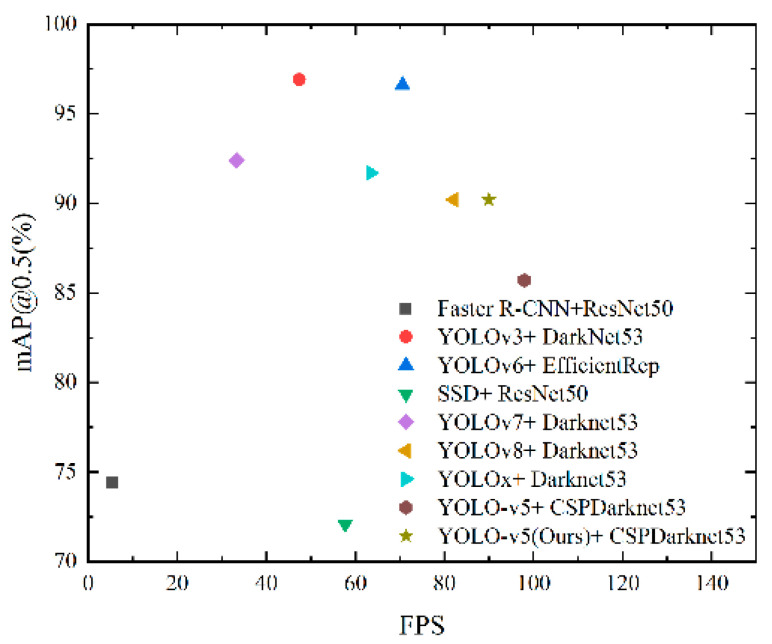
Performance comparison of different models.

**Figure 12:**
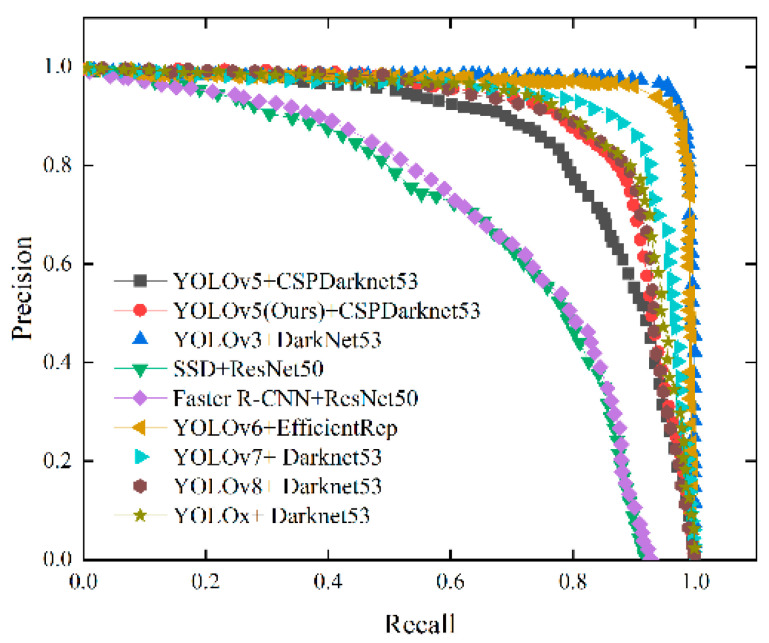
Precision–recall cures of different models.

After trained on 200 epochs using the same dataset, the improved YOlO5 algorithm was compared with algorithms such as YOLOv3, YOLOv6, YOLOv7, YOLOv8, and YOLOx for target detection accuracy, speed, and real-time target detection speed. The results are shown in Table 3, Figure 11 and Figure 12, respectively. The target detection accuracies of the improved YOLOv5 algorithm, YOLOv3, YOLOv6, YOLOv7, YOLOv8, and YOLOx are 90.2%, 96.9%, 96.6%, 92.4%, 90.2%, and 91.7%, respectively. Yet, the improved YOLOv5 algorithm is one of two models in Table 3 that exceed 90 FPS, while the FPS of other models are far lower, indicating that the improved YOLOv5 algorithm has better real-time detection performance. The improved YOLOv5 algorithm and the YOLOv5 algorithm have the smallest Params and GFLOPs from a lightweight point of view, as is shown in Table 3. As Figure 12 shows, the YOLOv3, YOLOv6, YOLOv7, YOLOv8, YOLOx, and improved YOLOv5 algorithms perform better than the original YOLOv5 algorithm in terms of target detection accuracy.

Overall, although the accuracy of the YOLOv3, YOLOv6, YOLOv7, and YOLOx algorithms is higher than that of YOLOv5, their Params and GFLOPs are far higher than YOLOv5, meaning they cannot meet the real-time and lightweight requirements. Meanhile, the FPS of the improved YOLOv5 algorithm and YOLOv5 are above 90, meaning they can meet the real-time requirement, while the accuracy of the improved algorithm is much better than that of YOLOv5. So, the improved algorithm achieves the best overall performance.

## 7. Conclusions

The improved YOLOv5 algorithm was proposed in the paper, and its object detection performance was compared with those of the YOLOv3, YOLOv6, YOLOv7, YOLOv8, YOLOx, SSD, and Faster R-CNN algorithms for VRUs. The results of the comparative evaluation show that it has the best overall performance in terms of efficiency, accuracy, and timeliness.

The proposed algorithms have very fast detection speeds under the premise of guaranteeing the detection accuracy, meaning they can provide real and reliable environment perception information on vulnerable road users for the autonomous emergency braking technology.

The proposed future directions for improving our model mainly including two aspects. First, when the YOLOv5 algorithm formulates the deep learning target-detection dataset, it fails to cover the training images under scenarios such as rainy, snowy weather, and so on, resulting in a dataset that is not broad enough, and to some extent, this will constrain the accuracy of the algorithm and the application scenarios. In this regard, improvement of the dataset should be considered in subsequent research to further improve the performance of the algorithm. The second suggestion is that research on making deep neural networks lightweight should be carried out to reduce the network size based on the premise of ensuring recognition accuracy and detection speed.

## Figures and Tables

**Table 1:** Different datasets and their features.

Database Name	Database Features
Caltech Pedestrian Database [26]	The vehicle-mounted camera is used for pedestrian detection, where there are many pedestrian occlusion situations, close to real road conditions.
Daimler Database [27]	It is obtained using vehicle-mounted cameras and divided into detection and classification.
Tsinghua-Daimler Dataset [28]	It includes categories such as pedestrians, bicyclists, motorcyclists, tricyclists, and so on.
Cityscapes Dataset [29]	Contains stereo video sequences captured in 50 different city street scenes with dense pixel annotation in 19 categories.
Penn-Fudan Database [30]	The dataset, co-produced by the University of Pennsylvania and Fudan University, is based on scenes from campus and in the city and provides a good representation of the Chinese pedestrian characteristics.
NuScenes Dataset [31]	A large-scale 3D autonomous driving dataset built by nuTonomy, in which the test vehicle is equipped with one LIDAR, five long-range radars, and six vision sensors covering complex urban scenes with rich data annotation.

**Table 2:** Algorithmic experimental environment.

Software and Hardware Categories	Parameters	Software and Hardware Categories	Parameters
Operating System	Windows 10	GPU	NVIDIA RTX 3060
CPU	AMD Ryzen 7 5800H	Memory	16GB DDR4 3200MHz
GPU Acceleration Framework	CUDA 11.3	Programming Platform	PyCharm 2021.3
Programming Languages	Python 3.8	Deep Learning Framework	PyTorch 1.10

**Table 3:** Performance evaluation of object detection in the same dataset using different algorithms.

Method	Backbone	mAP@0.5 (%)	FPS	Params(M)	GFLOPs
Faster R-CNN [6]	ResNet50	74.4	5.3	42.01	19.90
YOLOv3 [15]	DarkNet53	96.9	47.4	61.53	193.89
YOLOv6 [32]	EfficientRep	96.6	70.6	17.19	44.12
SSD [8]	ResNet50	72.1	57.7	26.02	34.8
YOLOv7	Darknet53	92.4	33.3	36.49	103.50
YOLOv8	Darknet53	90.2	82.1	43.63	165.42
YOLOx	Darknet53	91.7	63.4	8.94	26.64
YOLOv5 [19]	CSPDarknet53	85.7	98	7.02	15.80
YOLOv5(Ours)	CSPDarknet53	90.2	90	7.64	16.70

## Data Availability

Not applicable.
